# Presentation of 493 Consecutive Girls with Idiopathic Central Precocious Puberty: A Single-Center Study

**DOI:** 10.1371/journal.pone.0070931

**Published:** 2013-07-30

**Authors:** Eloïse Giabicani, Slimane Allali, Adélaïde Durand, Julie Sommet, Ana-Claudia Couto-Silva, Raja Brauner

**Affiliations:** Université Paris Descartes and Fondation Ophtalmologique Adolphe de Rothschild, Paris, France; John Hopkins University School of Medicine, United States of America

## Abstract

**Background:**

Despite the number of reported data concerning idiopathic central precocious puberty (CPP) in girls, major questions remain including its diagnosis, factors, and indications of gonadotropin releasing hormone (GnRH) analog treatment.

**Methods:**

A retrospective, single-center study was carried out on 493 girls with CPP.

**Results:**

Eleven girls (2.2%) were aged less than 3 years. Breast development was either isolated (Group 0, n = 99), or associated with one sign, pubic hair development, growth rate greater than 2 standard deviation score (SDS) or bone age (BA) >2 years above chronological age, (Group 1, n = 187), two signs (Group 2, n = 142) or three signs (Group 3, n = 65). The interval between onset of puberty and evaluation, body mass index (BMI) SDS, plasma luteinising hormone (LH) concentrations (basal and peak) and LH/ follicle-stimulating hormone (FSH) peak ratio after GnRH test, plasma estradiol and uterus length were significantly greater in Groups 2 and 3 than in Groups 0 and 1 respectively. 211 (42.8%) patients were obese and/or had excessive weight gain during the year before puberty. Obese girls more often had BA advance of >2 years (p = 0.0004) and pubic hair development (p = 0.003) than the others. BMI did not correlate with LH or with LH/FSH peak ratio. Girls with familial history of early puberty (41.4%) had greater frequencies of pubertal LH/FSH peak ratios (p = 0.02) than the others. During the 31 years of the study, there was no increase in the frequency of CPP or variation in its characteristics.

**Conclusion:**

Obesity is associated with a higher BA advance and higher frequency of pubic or axillary hair development but not with LH secretion, suggesting that obesity accelerates adrenarche but not the maturation of the hypothalamic-pituitary-ovarian axis. The LH/FSH peak ratio was more frequently pubertal in girls with a familial history of early puberty, suggesting that this maturation depends on genetic factors.

## Introduction

Precocious puberty in girls is defined by the development of sexual characteristics before the age of 8 years [1]. Precocious breast development is usually due to the premature activation of the hypothalamic-pituitary-ovarian axis, known as central precocious puberty (CPP), and not to primary ovarian or adrenal causes. It may also correspond to premature thelarche (PT), which is defined by non-pathological isolated early breast development, generally during the first two years of life, but progression from PT to CPP occurs in 13% of cases [2], [3]. This finding led to the hypothesis that PT and CPP may represent different positions along a continuum of hypothalamic gonadotropin releasing hormone (GnRH) neuron activation [4].

American guidelines propose that girls who develop breasts or pubic hair before the age of 7 (White girls) or 6 years (African-American girls) should be evaluated [5]; however in Europe the limit was maintained at 8 years because pubertal changes between 6 and 8 years may be secondary to hypothalamic-pituitary lesion [6], [7]. Four series recently reported the frequency of CPP in children referred for signs of early puberty [8]–[11].

In girls, CPP is idiopathic in the majority of cases. The factors contributing to the earlier onset of puberty are genetic [9] and/or environmental, particularly obesity [12]–[15].

Despite the number of reported data and analyses concerning idiopathic CPP in girls, major questions remain, including 1) the differential diagnosis between CPP and PT, including the interpretation of hormonal test results that support the diagnosis, 2) the respective role of genetic and environmental factors, and 3) the evolution of estrogen secretion and indications of GnRH analog treatment [16], [17].

We studied a cohort of 493 consecutive girls followed by the same physician for idiopathic CPP. We analyzed the CPP clinical and biological presentation and the factors influencing it; we detailed the presentation of girls with puberty onset before 3 years. We also determined CPP evolution over time (31 years) by determining the annual number of CPP cases and the respective frequencies of patients aged less than 6 years or having a familial history of early puberty or increased body mass index (BMI). We expect this analysis to facilitate the diagnosis, follow-up and treatment decisions concerning these girls.

## Methods

### Ethics statement

Written informed consent for the evaluations was obtained from the children's parents and included in the children's hospital medical record. The Ethical Review Committee (Comité de Protection des Personnes, Ile de France III) approved this retrospective study and stated that “This study appears to be in accordance with the scientific principles generally accepted and to the ethical standards of research. The study was lead in the respect of the French law and regulation”.

### Patients

This retrospective single-center cohort study was carried out in 493 consecutive girls monitored for idiopathic CPP by a senior pediatric endocrinologist (R Brauner) in a university pediatric hospital from June 1981 to July 2012, including some who were previously reported [18], [19]. We did not include 47 other girls who were first evaluated elsewhere (n = 2), observed for a second opinion (n = 1) and those who didn't undergo GnRH stimulation test (n = 44). CPP was diagnosed on the appearance (confirmed by palpation) of breast development before the age of 8 years accompanied by the presence of pubic or axillary hair, a growth rate greater than 2 SD scores (SDS) the year before clinical evaluation (health records data) and/or a bone age (BA) more than 2 years above chronological age [20]. The gonadotropins response to the GnRH stimulation test was not used as criterion for inclusion [1]. We also considered as CPP, not PT, 99 girls for whom at presentation, breast development was clinically isolated but associated with pubertal uterus length, luteinising hormone (LH)/follicle-stimulating hormone (FSH) peak ratio and/or plasma estradiol concentrations (see Methods) or for whom the clinical picture of CPP became complete before 8 years of age.

The patients were classified according to the number of the above signs of puberty (presence of pubic or axillary hair, a growth rate greater than 2 SDS and/or BA more than 2 years above chronological age) [20] associated with breast development at evaluation: isolated breast development (group 0) and associated with one sign (group 1), two signs (group 2) or three signs (group 3) [4].

Organic intracranial lesions were excluded by neuroradiological evaluation (computed tomography scan or magnetic resonance imaging (MRI) in the more recent patients), as were ovarian [21] and adrenal disorders [22]. Neuroradiological evaluation was not performed in 57 (11.6%) girls who had a familial history of early puberty, normal neurological evaluation, were aged more than 6 years at onset of puberty and had low plasma estradiol concentrations (<15 pg/ml) [6]. The plasma 17-hydroxyprogesterone and testosterone concentrations were measured in girls with pubic or axillary hair development to exclude abnormal androgen secretion and congenital adrenal hyperplasia [22]. Plasma thyroxin and thyroid-stimulating hormone concentrations were measured to exclude hypothyroidism, and 24 h urinary cortisol was measured to exclude hypercortisolism in those who were overweight or had a rapid weight increase.

### Methods

Familial history of early puberty was defined as the mothers undergoing menarche (data available in 418 cases) before the age of 11 years and/or the presence of an early onset of puberty in the father, brother, sister, or grandparents [9]. The initial evaluation included determinations of height, growth rate, weight, pubertal stage and BA and evaluation of the hypothalamic-pituitary-ovarian axis by measuring basal and GnRH (100 μg/m^2^; maximum dose 150 μg)-stimulated LH and FSH peaks and plasma concentrations of estradiol. Age at first pubertal sign was reported by the parents and interval-times between puberty onset and first examination were calculated.

Height, growth rate and BMI (weight in kg/height in m squared) were expressed as SDS for chronological age [23], [24]. Obesity was defined as BMI over 2 SDS. The pubertal stage was rated according to *Marshall and Tanner* [25]. BA was assessed (by R Brauner) using the *Greulich and Pyle* method [26]. Plasma LH, FSH and estradiol concentrations were measured with various RIAs during the study period. When the assay method for a given hormone was changed, it was cross-correlated with the previous method. Thus, the results for a given parameter were comparable throughout the entire period. The following values were considered to be pubertal: uterus length ≥35 mm [27], LH/FSH peak ratio after GnRH test ≥0.66 [28], and plasma estradiol concentrations ≥15 pg/mL (55 pmol/L).

The data were expressed as the means ± SD. Statistical analyses were performed with Graph Pad Prism 6 using non parametric tests (Wilcoxon-Mann-Whitney, Fisher's, χ^2^, and Spearman's correlation).

## Results

The age at onset of puberty was <3 years in 11 (2.2%) patients, 3–6 years in 76 (15.4%), 6–7 years in 111 (22.5%) and 7–8 years in 295 (59.8%). The interval between the onset of puberty and the initial evaluation was 0.9±0.8 (0–6) years. Twenty-seven (5.5%) girls were adopted, and one was born after an egg donation.

### Presentation

The characteristics of the patients and the percentages of increased values at the initial evaluation are shown in Table 1.

**Table 1:** Characteristics of 493 girls with idiopathic CPP.

	n	Mean ± SD	Min	Max	Remarkable values		
						n	%
**Age at onset, years**	493	6.68±1.35	0.10	8.0	<6	88	17.8
**Age at evaluation, years**	493	7.55±1.44	0.80	10.6	-	-	-
**Growth rate the year before, SDS**	444	2.27±2.29	−4.60	12.9	≥2	208	46.8
**Weight gain the year before, SDS**	395	1.94±2.03	−2.26	14.13	≥2	153	38.6
**BMI at first evaluation, SDS**	493	1.28±1.56	−2.40	6.6	≥2	136	27.6
**BA advance, years**	478	1.25±1.25	−2.50	6.0	≥2	145	30.3
**Uterus length, mm**	270	35.99±8.81	16.00	75.0	≥35	149	55.2
**Estradiol, pg/mL**	479	16.24±17.3	0.04	177.0	≥15	162	33.8
**LH peak, IU/L**	493	11.19±14.37	0.20	101.0	-	-	-
**FSH peak, IU/L**	493	12.62±6.83	0.80	62.0	-	-	-
**LH/FSH peak ratio**	493	0.95±1.12	0.04	9.8	≥ 0.66	224	45.4

The characteristics of the 11 girls who began puberty before 3 years are shown in Table 2. The LH/FSH peak ratio and the plasma estradiol concentrations were prepubertal in 5 of these girls, but pubertal development progressed before 8 years in all, leading to treatment with GnRH analogs in all but case 5. Six out of 11 had familial history of early puberty.

**Table 2:** Characteristics of 11 girls who began idiopathic CPP before 3 years.

	1	2	3	4	5	6	7	8	9	10	11	Mean
Age at onset, years	0.1	0.5	0.7	1	1.0	1.3	1.7	1.8	2.0	2.0	2.5	1.3
Age at evalu ation, years	1.0	0.8	0.8	3.0	1.1	1.4	2.0	2.3	2.7	2.3	2.6	1.8
Height, SDS	1.6	1.3	−0.8	2.6	0.8	0.8	2.5	0.8	2.2	4.3	0.2	1.5
BMI, SDS	1.9	−1	−0.9	0.1	−0.6	−1	−0.7	0.1	4.7	0.1	−1	0.2
Tanner stage	B3P2	B2P2	B2P2	B3P2	B2P3	B3P1	B2P1	B2P1	B3P2	B2P1	B2P1	-
Bone age ad vance, years	1.9	0.0	0.2	4.0	-	0.0	3.3	0.7	1.5	3.0	-	1.1
Growth rate, SDS	-	-	-	2.3	-	-	2.3	0.1	0.1	7.8	0.1	2.1
LH peak, IU/L	2.5	3.1	52.0	34	0.2	7.70	17.4	2.6	23.0	36.5	42.0	20.1
FSH peak, IU/L	24.0	28.0	27.0	23.3	4.4	21.0	11.4	17.0	21.4	9.3	16.5	18.5
LH/FSH peak ratio	0.1	0.11	1.93	1.46	0.05	0.37	1.53	0.15	1.07	3.92	2.55	1.2
Estradiol, pg/mL	10.0	10.0	100.0	50.0	17.0	10.0	30.0	9.0	15.0	38.0	33.0	29.3
Uterus length, mm	-	27	-	-	26	26	-	-	49	45	-	34.6
Familial CPP	yes	yes	no	no	yes	yes	no	no	yes	yes	no	-
GnRH analogue treatment/ age at introduction	yes/6.5	yes/?	yes/0.9	yes/5.0	no	yes/8.3	yes/2.1	yes/4.4	yes/2.9	yes/2.4	yes/2.7	-
Age at first menstruation, years	11.8	9.2	-	12.7	-	-	12	13.5	8.4	10.0	-	11.6
Final height, SDS	0.7	-	-	−0.8	-	-	1.4	1.0	−0.9	-	-	0.3

Breast development was the first sign of puberty in 341 (69.2%) girls. At the evaluation, it was present in all girls and was stage 2 in 222 (45.0%) girls and greater in the others. Menstruation before any other clinical presentation occurred in only two (0.4%) girls at 7.4 and 7.9 years. Breast development was accompanied by pubic or axillary hair development in 313 (63.8%) patients, a growth rate greater than 2 SDS in 208 (46.8%), and/or a BA greater than 2 years above chronological age in 145 (30.3%). These features were absent and breast development was the only presentation in 99 (20.1%) girls. Among these girls, 75 presented with breast development associated with pubertal uterus length ≥35 mm (n = 23), LH/FSH peak ratios ≥0.66 (n = 34), and plasma estradiol concentrations ≥15 pg/mL (n = 18). In the remaining 24, the previous features were absent, and the breast development was isolated at presentation, but the clinical picture of CPP became complete before 8 years of age. The next presenting sign of puberty was pubic hair development, growth rate increase and/or BA progression, leading to GnRH analog treatment in 5 of them.

**Figure 1:**
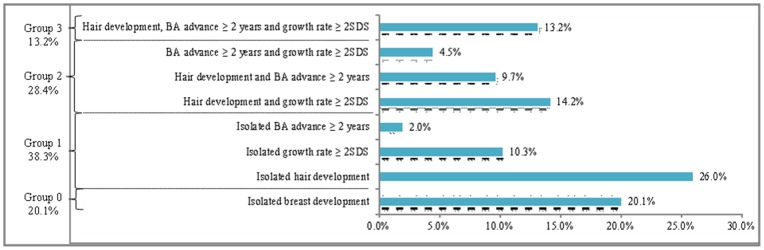
Distribution of the 493 girls with idiopathic CPP classified according to the number of the signs of puberty associated with breast development.

The patients were classified according to the number of the above signs of puberty associated with breast development at evaluation (Fig 1): isolated breast development (group 0, n = 99) and associated with one sign (group 1, n = 187), two signs (group 2, n = 142) or three signs (group 3, n = 65). Groups 0 and 1 showed similar characteristics, as did groups 2 and 3. The 4 groups were not statistically different with respect to target height, familial history of early puberty, age at onset of puberty (mean and percentage of those younger than 6 and 7 years respectively), and anterior pituitary height on MRI, but they differed significantly in terms of interval between the onset of puberty and evaluation, BMI SDS, weight gain the year before puberty onset, plasma LH concentrations (basal and peak) and LH/FSH peak ratios after GnRH test (means and percentage of pubertal values), plasma estradiol concentration and uterus length (Table 3). Group 3 girls were older than those of group 0 at the initial evaluation (p = 0.02) and were more frequently treated with GnRH analogs (56.9%) compared with girls in groups 0 (34.7%, p = 0.006) and 1 (33.7%, p = 0.001). In total, 43.7% of girls in group 2 were treated with GnRH analogs. The frequency of obese girls (BMI >2 SDS) was significantly lower in group 0 (14.1%) compared to group 1 (25.7%, p = 0.03), group 2 (33.8%, p = 0.0006) and group 3 (40.0%, p = 0.0003). Group 1 girls were also significantly less obese compared to group 3 (p = 0.04).

**Table 3:** Comparison in 493 girls with idiopathic CPP classified according to the number of signs of puberty associated with breast development (See Fig 1).

Interval- time, years	0	1	2	3	LH/FSH peak ratio	0	1	2	3
	0.7±0.6*	0.9±1.0	0.9±0.6	1.0±0.6		0.81±1.3	0.77±0.8	1.16±1.1	1.24±1.4
**0**		NS	P = 0.007	p = 0.0004	**0**		NS	P<0.0001	p = 0.0007
**1**			NS	p = 0.03	**1**			p<0.0001	p = 0.0008
**2**				NS	**2**				NS

BMI, SDS	0	1	2	3	LH/FSH peak ratio >0.66 , %	0	1	2	3
	0.75±1.2	1.11±1.5	1.64±1.6	1.85±1.6		34.3	39.6	54.2	60.0
**0**		NS	p<0.0001	p<0.0001	**0**		NS	p = 0.003	p = 0.001
**1**			p = 0.006	p = 0.003	**1**			p = 0.01	p = 0.006
**2**				NS	**2**				NS

LH basal, IU/L	0	1	2	3	Estradiol, pg/mL	0	1	2	3
	0.65±2.2	0.62±0.9	1.09±1.3	1.08±1.8		11.93±12.6	13.36±13.1	19.04±17.2	24.69±27.5
**0**		NS	p<0.0001	p = 0.02	**0**		NS	p = 0.002	p<0.0001
**1**			p<0.0001	NS	**1**			p = 0.02	p = 0.0002
**2**				NS	**2**				NS

LH peak, IU/L	0	1	2	3	Uterus length, mm	0	1	2	3
	8.09±9.6	9.56±11;7	14.76±17.0	16.52±18.2		34.3±9.7	34.4±7.4	38.7±9.4	38.9±9.1
**0**		NS	p = 0.0005	p = 0.001	**0**		NS	p = 0.007	NS
**1**			p = 0.0006	p = 0.003	**1**			p = 0.002	NS
**2**				NS	**2**				NS

### Plasma LH and FSH

The plasma basal LH and FSH concentrations and their response to the GnRH test were analyzed. The LH/FSH peak ratio was pubertal (≥0.66) in 224 (45.4%) girls. The basal LH level correlated positively with the LH peak (ρ = 0.60, p<0.0001) and the peak ratio (ρ = 0.58, p<0.0001). The peak LH level also correlated positively with the peak ratio (ρ = 0.89, p<0.0001). All but 4 of the 59 girls with a basal LH level over 1.2 IU/L had a pubertal LH/FSH peak ratio (sensitivity 31.6%, specificity 98.2%, and positive predictive value 93.2%). The LH/FSH peak ratio (< or ≥0.66) was compared to the LH peaks with various thresholds that were previously used to define a pubertal response to the GnRH test (>5, n = 274; >15, n = 126; and >25, n = 62, IU/L): the sensitivity and specificity were 77% and 95% for a limit of 5 IU/L, 54% and 99% for a limit of 15 IU/L, and 27% and 99% for a limit of 25 IU/L, respectively. The LH concentrations (basal and peak) and the LH/FSH peak ratio did not correlate with the age at onset of puberty, interval between the onset of puberty and the evaluation, BMI, weight gain the year before puberty onset (SDS), BA advance, uterus length, plasma estradiol concentration, or anterior pituitary height on MRI.

### Factors of presentation

136 (27.6%) patients were obese at evaluation. Furthermore, the weight gain the year before puberty onset is increased (1.9±1.9, [−2.7 to 6.7] SDS) in the 396 girls (80%) for whom we had information. After the exclusion of obese patients, 75 patients had excessive (>2 SDS) weight gain the year before puberty onset. Thus, at least 211 (42.8%) patients were obese and/or had excessive weight gain during the year before puberty onset. Obese girls more often had a BA advance of 2 years and more (p = 0.0004) and pubic or axillary hair development (p = 0.003) than the other girls, but they showed no differences in the growth rate, LH/FSH peak ratio, plasma estradiol concentration, uterus length, or frequency of familial history of early puberty. There was no correlation between BMI and the age at puberty onset, BA advance, plasma estradiol concentrations, LH (basal and peak values), LH/FSH peak ratio, or uterus length. Additionally, no correlation was found between plasma estradiol concentrations and the above characteristics.

The growth rate did not correlate with any of the characteristics of CPP.

When girls aged less than 6 years at onset of puberty were compared to older girls, they had significantly lower BMIs SDS (p = 0.007) and lower frequencies of pubic hair development (p = 0.02), values of the pubertal LH/FSH peak ratio (p = 0.04), and lower uterus length (p = 0.03). However, they did not differ in the other characteristics or frequency of indications for GnRH analog treatment.

When girls with a familial history of early puberty (n = 192, 41.4% on 465 after exclusion of the adopted) were compared to other girls, as expected, their mothers had their first menstruations younger (p = 0.0001), and they had significantly greater frequencies of pubertal LH/FSH peak ratios (p = 0.02), lower plasma estradiol concentrations (p = 0.0001) and a greater plasma uterus length (p = 0.03). However, they did not differ in the other characteristics or frequency of indications for GnRH analog treatment.

When girls with pubertal values of the LH/FSH ratio were compared to those with prepubertal values, they were more often aged more than 6 years (p = 0.0002) and had a familial history of early puberty (p = 0.02), BA advance greater than 2 years (p<0.0001), a growth rate higher than 2 SDS (p = 0.006), an LH peak higher than 5 IU/L (p<0.0001), higher plasma estradiol concentrations (p<0.0001), pubertal uterus length (p<0.0001), and GnRH analog treatment indications (p<0.001). There was no difference in the BMI.

### Evolution over time

**Figure 2:**
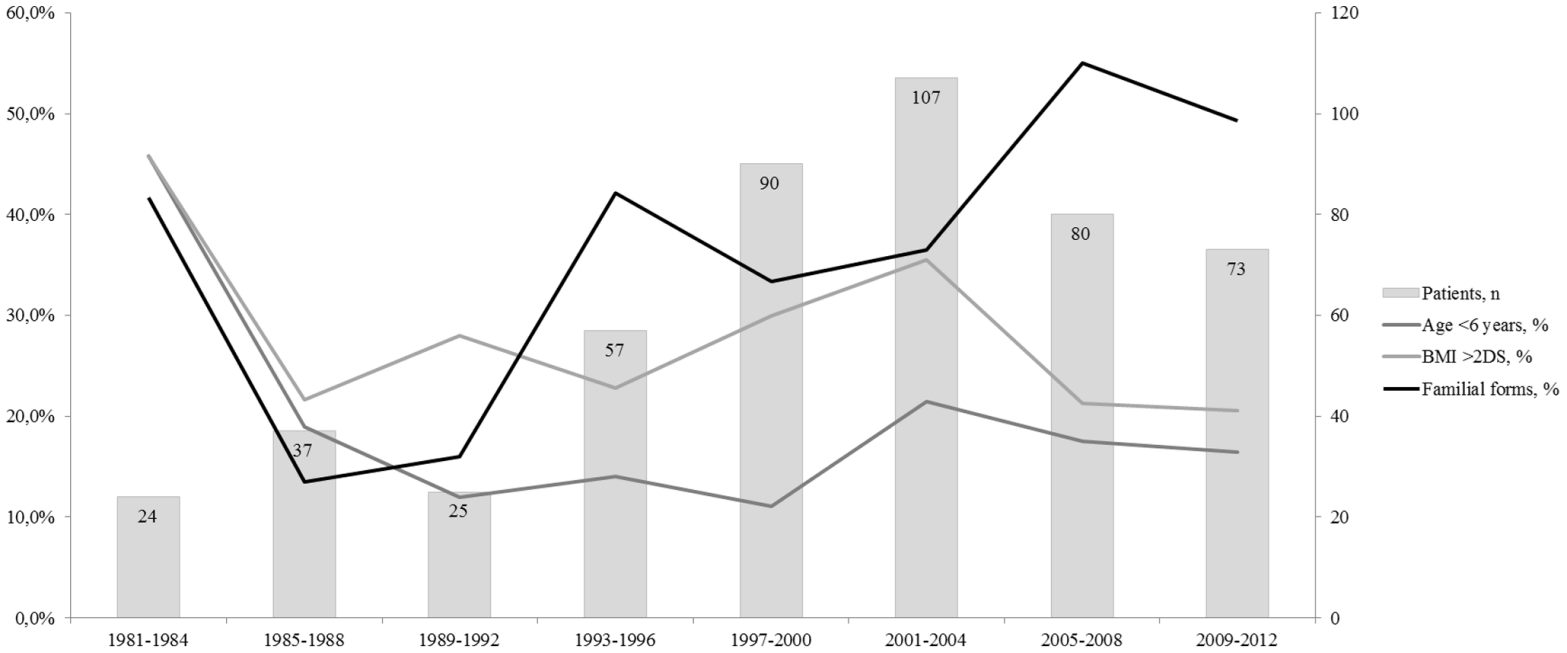
Evolution of the numbers and characteristics over 31 years of 493 girls with idiopathic CPP.

There was a slight augmentation in the annual number of girls observed for idiopathic CPP at the end of the nineties, followed by stabilization in the last 10 years, with a mean of 29 per year (Fig 2). The proportion of girls aged less than 6 years with CPP did not change over the 31 years of the study period in this population recruited in the same geographical area. There was a slight increase in the percentage of familial forms. The BMI remained stable during this period, with some marginal significant difference but no significant variation over time.

## Discussion

The main results of this study are 1) the increase in the number of the signs associated with breast development at CPP presentation is associated with a significantly higher BMI SDS, frequency of obesity, LH basal and peak levels, LH/FSH peak ratios, and plasma estradiol concentrations; 2) obesity is associated with a higher percentage of BA advance greater than 2 years and a higher frequency of pubic or axillary hair development but not with LH secretion, suggesting that obesity accelerates adrenarche but not the maturation of the hypothalamic-pituitary-ovarian axis. The BMI SDS and the frequency of obesity in girls with CPP are significantly greater than in the general population, but there was no increase in these parameters over the 31 years of the study. During this period, we found no increase in the frequency of CPP or variation in its characteristics (BMI SDS or proportion of girls aged less than 6 years).

### Presentation

The distribution of the ages at onset of puberty is similar to that reported in a multicenter Italian study on 428 girls with CPP, including 56 with the organic form [29]. Only 2.2% (n = 11) of the girls were aged less than 3 years at the onset of puberty. We found no specific reports on CPP before this age. Six of these cases had a familial history of early puberty. All of these patients except one had rapidly progressing CPP requiring GnRH analog treatment at the first evaluation or during the follow-up. This population is most likely important to identify the genes implicated in CPP and, more generally, pubertal development [30]. Their presentation was also different from girls with ovarian cysts; indeed, girls with ovarian cysts are frequently aged less than 3 years, but they present with menstruation and low plasma LH and FSH concentrations without an increase after the GnRH test [21]. None of the 5 aged less than 3 years evaluated by ultrasonography had ovarian cyst. At the other extremity, 60% of the girls began puberty between 7 and 8 years. Among these, 47.2% had a familial history of early puberty. This population underlines the problem of hypothalamic-pituitary lesion investigation and GnRH analog treatment indication. We found that low plasma estradiol concentrations at this age suggest lower chance of a lesion [6], but this finding was not confirmed by a recent large study [7]. Among the 30 girls with precocious puberty associated with a hypothalamic-pituitary lesion we reported recently [31], 10 were aged 7 to 8 years at breast development due to hypothalamic hamartoma in 2 cases and optic glioma in 8.

The patients were classified according to the number of signs associated with breast development at first evaluation. Patients with isolated breast development did not differ from those having one associated sign but differed from those with two or three signs. Although the interval between the onset of puberty and evaluation was slightly but significantly greater in patients with multiple associated signs, which suggested that this factor may be partly responsible for the presentation, complete presentation was associated with greater BMI, an increase in weight the year before the puberty onset, plasma LH basal concentrations and the LH response to the GnRH test, estradiol concentration and uterus length. As patients with obesity and/or increased weight had significantly greater BA advance and hair development but not different LH concentrations (basal and response to the GnRH test), obesity may accelerate adrenarche but not the maturation of the hypothalamic-pituitary-ovarian axis. In a previous study on 115 girls with CPP (who were also included in the present study) evaluated for dehydroepiandrosterone sulfate (DHAS), which is the marker of adrenarche, those with a lower BMI had a lower DHAS than those with a BMI greater than one SDS [19]. In a previous study on children with premature pubarche, we showed that the plasma DHAS concentrations were higher in obese girls than in lean ones (p = 0.009), with a positive correlation between DHAS and BMI z-score (p = 0.004) [32]. This correlation persisted after adjustment for the time from the onset of premature pubarche to evaluation and the Tanner stage. Shirtcliff et al. [33] showed the good correlation between androgens levels and Tanner stage for hair development, which support our hypothesis. Furthermore, Neville et al. [34] found that 30% of children with premature pubarche were obese.

### Plasma LH and FSH

The plasma basal LH and FSH concentrations and their response to the GnRH test were analyzed. We used an LH/FSH peak ratio after GnRH test ≥0.66 [28] as the gold standard of the maturation of the hypothalamic-pituitary-ovarian axis and tested various limits of the LH peak. These authors [28] showed that this ratio or an LH peak ≥15 IU/L detected 96% of pubertal girls with no false positives. We found that 99% of the girls with an LH peak ≥15 IU/L had a pubertal ratio, but 54% of those with a pubertal ratio had an LH peak <15 IU/L. The limit of 5 IU/L used to define CPP [1] was more effective in terms of specificity (77%), with a good sensitivity ( 95%). We were surprised to find that the LH concentrations (basal and peak) and LH/FSH peak ratio were not correlated with any of the criteria of CPP in the entire population. They were significantly increased with the number of signs of puberty associated with breast development at the evaluation. LH plasma concentrations (basal and response to the GnRH test) seem to be independent of CPP characteristics. The ratio was more frequently pubertal in girls with a familial history of early puberty. This result suggests that the maturation of the hypothalamic-pituitary-ovarian axis depends on genetic factors. We have previously shown that a lower LH/FSH peak ratio in untreated girls with CPP is associated with a smaller height loss, as evaluated by the difference between the height at initial evaluation and the predicted adult height [35]. The factors associated with adult height of the girls in this study, either treated with GnRH analogs or not, are under evaluation.

### Evolution over time

There was a slight but not significant augmentation in the annual number of girls observed for idiopathic CPP over the 31 years. The BMI remained stable during this period. This study is limited by the fact that all patients were observed by a single physician. However, it allows standardized recruitment procedures and all patients with CPP diagnosis were included.

Teilmann et al. [36] reported that 670 children with precocious pubertal development were diagnosed with precocious puberty from 1993 to 2001, corresponding to 50 to 70 new cases per year in Denmark, with a constant incidence during the study period. The same team [37] later reported that among Danish girls, the onset of puberty, defined as the mean estimated age at attainment of glandular breast tissue, occurred significantly earlier in 2006 compared with 1991. Earlier breast development was not associated with higher levels of gonadotropins, a sign of earlier pubertal activation of the pituitary-gonadal axis. In Denmark, the prevalence of overweight and obese children has also increased. However, the authors did not find any difference in the BMI of the 2 cohorts of girls, and adjusting their data on puberty timing for BMI did not change the results. They concluded that the increasing incidence of obesity among children could not explain their findings and suggested that increased exposure to endocrine-disrupting chemicals from a modern lifestyle may be involved in the observed trends. The difference between induced PT and true precocious puberty is important regarding these potential exogenous factors.

## Conclusion

This study suggests for the first time that obesity accelerates adrenarche but not the maturation of the hypothalamic-pituitary-ovarian axis. Why is obesity associated with premature pubarche in certain girls and CPP in others? What is the role of intrauterine growth retardation? Does hypothalamic-pituitary-ovarian axis maturation depend mainly on genetic factors, which is suggested by the LH/FSH peaks ratio more frequently pubertal in girls with a familial history of early puberty, and not obesity? What is the relationship with adrenarche?

The pubic hair development and part of the BA advance observed in CPP express the adrenarche and are linked with obesity. Half of the girls reached their adult height after spontaneous growth or treatment with GnRH analogs. The factors associated with adult height, primarily the role of adrenarche and hypothalamic-pituitary-ovarian axis activation, are under evaluation.
